# Formulation and Process Optimization of *Rauvolfia serpentina* Nanosuspension by HPMC and In Vitro Evaluation of ACE Inhibitory Potential

**DOI:** 10.3390/jfb13040268

**Published:** 2022-11-30

**Authors:** Syeeda Iram Touqeer, Nazish Jahan, Naseem Abbas, Ahsan Ali

**Affiliations:** 1 Natural Products Lab, Department of Chemistry, University of Agriculture, Faisalabad 38000, Pakistan; 2 Department of Mechanical Engineering, Sejong University, Seoul 05006, Republic of Korea; 3 Department of Mechanical Engineering, Gachon University, Seongnam-si 13120, Republic of Korea

**Keywords:** angiotensin converting enzyme, Hippuryl-histidyl-leucine, *Rauvolfia serpentina*, nanosuspension

## Abstract

Angiotensin converting enzyme (ACE) overactivation is one of the primary causes of hypertension, which leads to cardiovascular disorders all over the world. In the scientific world, nanosuspension is a novel area of study that could offer an alternative treatment for active pharmaceuticals that are not well soluble in water. Since active compounds’ bioavailability is reduced by their poor solubility, there are eventually fewer applications. Drug solubility, dissolving rate, and bioavailability are improved by nanosuspension, which shrinks medication particle size into the nanoscale range and boosts the surface area to volume ratio of the drug. There is a need to prepare *Rauvolfia serpentina*’s nanosuspension in order to get around some of the major challenges that it faces because of its poor solubility and wide range of biological activities. Using the antisolvent precipitation approach, a nanosuspension of *Rauvolfia serpentina* was created with hydroxy propyl methyl cellulose (HPMC). *Rouvolfia serpentina* nanosuspensions were prepared using a design of expert (DOE) approach, which allowed for the evaluation of key process parameters. To get an optimal sample, the effects of stabilizer concentration and anti-solvent volume on particle size, zeta potential, and PdI using CCD-RSM were investigated. Using the substrate Hippuryl-histidyl-leucine, the in vitro ACE inhibitory potential was assessed. On human erythrocytes, the safety of nanosuspension was evaluated in vitro. The ideal value of independent variables was discovered to be 0.25% *w*/*v* in order to achieve the desired response. Using scanning electron microscopy, the morphology of optimized nanosuspension was discovered to be rod-shaped (SEM). Compared to nanoformulation, crude extract had higher ACE inhibitory potential (83.11%). Human erythrocytes were found to be unaffected by nano-sized particles.

## 1. Introduction

Angiotensin converting enzyme (ACE) overproduction-induced hypertension is the main risk factor for common cardiovascular diseases, which are a significant cause of morbidity and mortality globally. A glycosylated zinc dipeptidyl–carboxypeptidase called angiotensin converting enzyme works with the renin–angiotensin–aldosterone system to regulate arterial blood pressure and electrolyte balance (RAAS). It does this by turning the dormant decapeptide angiotensin-I into the active octapeptide vasoconstrictor angiotensin [1]. Therefore, inhibition of ACE has become a modern therapeutic target to treat hypertension. Widely used synthetic ACE inhibitors (ACEI) are Captopril, lisinopiril, and Enalapiril [2]. However, it is believed that these synthetic ACEI have some undesirable side effects such as angioedema, cough, skin rashes, taste disturbances, and renal failure [3]. The potential to use natural sources as functional foods to counteract the negative effects of synthetic ACEI has recently attracted the attention of researchers. Therefore, efforts have been made to address plant components such flavonoids, peptides, and phenolic compounds to decrease ACE activity and reduce the high blood pressure [4,5]. Different plants have been reported to inhibit ACE activity such as the “ginger rhizomes” [6], the “Legumes” [3], the “bell peppers” [1], the “Apple” [7], the “Kiwifruit” [8], and the “Oil Palm” [2] but no one has reported the use of *Rauvolfia serpentina* for ACE inhibition.

A plant called asrol, often referred to as sarpagandha or *Rauvolfia serpentina*, may be found in almost all tropical and subtropical regions of the world that are habitable. It is a member of the Apocyanaceae family and the Plumeroidae subfamily. *Rauvolfia serpentina* is important from a medical standpoint because its roots contain secondary metabolites such as flavonoids, N-containing indole alkaloids, and phenolics. Its root extract is effective in the treatment of diseases that are mostly linked with the central nervous system, such as hypertension, insomnia, epilepsy, psychosis, insanity, and schizophrenia [9]. Particularly, in case of treatment for hypertension, its reserpine, ajamlicine, serpentine, ajmaline deserpidine, and yohimbine alkaloids are used [10,11]. However, it is renowned fact that almost 40% active chemical entities (flavonoids, alkaloids, phytochemical) of plants failed in the development pipeline due to their low water solubility, low dissolution rate, and consequently low oral bioavailability [12,13]. Drug solubility issues have been solved in a variety of methods, but nanotechnology has received a lot of interest in the scientific community due to its wide range of applications and capacity to alter materials on a nanoscale. It is a new area of medicine that is anticipated to have important therapeutic effects.

Nanosuspension is the colloidal dispersion of drug particles in nanometer range particularly 1 to 1000 nm in the presence of surfactant or [14,15]. It decreases medication particle size to the nanoscale range and improves the solubility, rate of dissolution, and bioavailability of components that are not very water soluble. There are two ways to make nanosuspension: top-down and bottom-up, and they differ in terms of the manufacturing concept [16,17]. Top-down methodology used media milling and high-pressure homogenization to break up bigger drug particles, microparticles, and nanoparticles. In order to obtain stable nanosuspension, bottom-up technologies such as antisolvent precipitation technique, micro emulsion, and melt emulsification method based on the assembly approach from molecules to drug particles that are nano-sized have been established. Antisolvent precipitation method is comparatively simple and cost effective [15,18].

Keeping in view the aforementioned facts, in the current study *Rauvolfia serpentina* has been used to inhibit ACEI potential through nanosuspension. The effect of formulative parameters such as concentration of stabilizer and volume of antisolvent on responses (particle size, PdI, and zeta potential) were investigated through central composite design of response surface methodology. The optimal formulation was used to determine ACEI potential and hemolytic activity in comparison with crude extract.

## 2. Materials and Methods

Most of the chemicals and reagents were of analytical grade (Sigma Aldrich, St. Louis, MI, USA).

### 2.1. Extraction of Plant and Preparation of Nanosuspension

**Figure 1:**
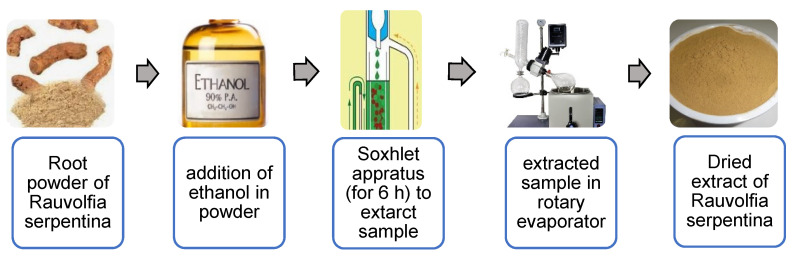
Flow chart showing extraction of plant material and preparation of nanosuspension.

Root powder (30 g) was extracted with ethanol (300 mL) on soxhlet apparatus for 6 h followed by solvent evaporation using a rotary evaporator as shown in Figure 1. *R. serpentina* nanosuspensions were prepared by anti-solvent precipitation method by following the procedure presented by He et al. 2015 [19], with some slight modification. Dry extract of *R. serpentina* was completely dissolved in ethanol to prepare organic phase. Distilled water containing HPMC act as an anti-solvent. A syringe was used to quickly inject organic solution into the antisolvent at a rate of 1 mL/min while stirring it vigorously for 6 h at a speed of 3000 rpm. Instantaneously, drug particles precipitated when two phases were mixed. Prepared nanosuspensions were filtered and poured into transparent bottles. Further, the prepared nanosuspensions were lyophilized before storage by using a lyophilizer (Beijing Sihuan Scientific Instruments Co., Ltd. Chongqing, China) to avoid aggregation and enhance the physical stability. Nanosuspensions were cooled at −18 °C for 12 h and then freeze-dried under vacuum (pressure < 10 pa) for 24 h.

### 2.2. Charecterization of Nanosuspension

#### 2.2.1. Particle Size and Zeta Potential

The dynamic light scattering zetasizer (DLS) was used to calculate the mean particle size and zeta potential (Zetasizer nano ZS, Malvern Instruments, Malvern, UK). Deionized water was used to dilute samples in order to combat the multi-scattering phenomenon. Each measurement was determined in triplicate at 25 °C with 633 nm laser wavelength, and average value was taken.

#### 2.2.2. Scanning Electron Microscopy

In order to visualize the shape of nanoparticles, optimized nanosuspension prepared with HPMC was scanned using scanning electron microscopy (SEM) (S-4800, Hitachi Technologies Corporation, Tokyo, Japan). For this, the sample was coated with gold by sputter coater and dried under vacuum. The SEM was implemented at an excitation voltage of 15 KV.

### 2.3. Optimization of Rauvolfia serpentina Nanosuspension

Design of expert (DOE) was used to minimize the number of experiments and to achieve optimum nanoformulation according to statistical calculations. Concentration of stabilizer (X_1_) and volume of antisolvent (X_2_) were selected as independent variables. The effect of these variables on particle size (Y_1_), zeta potential (Y_2_), and PdI (Y_3_) were studied due to their major contribution in formulated nanosuspension. Response surface methodology (RSM) was used to develop relationships among the above-mentioned parameters (independent and dependent). The range values for varying stabilizer concentration (0.2–0.30 g) and volume of anti-solvent (50–150 mL) were used to obtain the optimized model in Design Expert (DOE).

### 2.4. In Vitro Biological Activities

#### 2.4.1. ACE Assay

**Figure 2:**
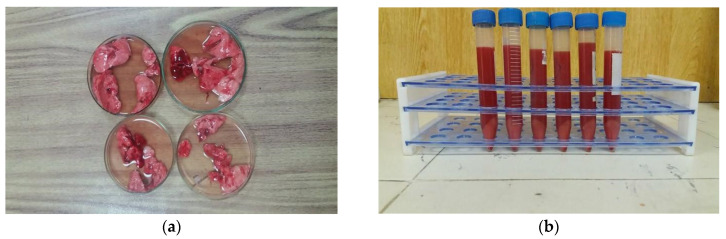
Preparation of acetone powder using fresh rabbit lung (**a**) through centrifugation (**b**).

ACE activity was assessed by following the procedure represented by Belovic et al. 2013 [20] with slight modification. Lungs from freshly slaughtered rabbits, as shown in Figure 2a, were washed with autoclaved saline solution (0.8%). Lung tissue was centrifuged for 10 min at 4000 rpm after being ground in sterile phosphate buffer saline solution as shown in Figure 2b. The supernatant was removed, and the residue was constantly cleaned with acetone over the course of an overnight. Filtration was used to remove the acetone, and the leftovers were dried out in the shade and then stored at 4 °C as refined lung acetone powder. Following that, borate buffer (10 mL, 100 mM, pH 8.3) was combined with lung acetone powder (0.5 g), and the mixture was continuously stirred for 12 h using a magnetic stirrer. The solution was then centrifuged for 45 min at 4000 rpm. The supernatant was dialyzed for two to three days with the aid of a dialyze membrane and additional borate buffer. The dialyzed solution was lyophilized and stored at −18 °C to produce ACE powder.

*Determination of ACE Activity:* ACE activity was determined by mixing ACE with borate buffer (10 mL of 100 mM, pH 8.3) to prepare ACE solution. ACE solution (50 µL) was mixed with borate buffer (50 µL) and incubated at 37 °C for 10 min. Substrate (150 µL, 8.3 mM of Hipl-His-Leu prepared in buffer) was added to the aforementioned solution, and the mixture was then incubated at 37 °C for 80 min. In order to inhibit the reaction between the ACE and substrate, 250 L of 1 M HCl was added. The prepared hippuric acid was extracted by adding 3 × 500 µL of ethyl acetate and centrifuged at 4000 rpm for 45 min. Supernatant (750 µL) was dried under ambient room conditions. A spectrophotometer was used to measure the absorbance at 228 nm after dried hippuric acid was dissolved in 1 mL of deionized water. The identical procedure was used to create the reaction blank; the only difference was the reagents’ adding sequence (HCl added before substrate). ACE activity was determined by following formula,

$$\mathrm{ACE}\;\mathrm{activity}=\left[A_{228}-A_{228\;\left(\mathrm{Blank}\right)}\right]\;$$

*ACE inhibitory potential:* The ACE inhibitory potential of crude, optimized nanosuspension, and captopril was determined using the aforementioned approach, but only after substituting the same volume of sample (1 mg/1 mL) for the borate buffer (50 L). The only thing that differentiated the creation of the reaction blank from the sample blank was the use of the tested sample in place of the borate buffer. %IACE was calculated by the following formula,

$$\%\mathrm{IACE}=100\frac{\left(A-B\right)-\left(C-D\right)}{\left(A-B\right)}$$

A = Absorbance in the presence of ACE

B = Absorbance of reaction blank

C = Absorbance in the presence of ACE and inhibitor

D = Absorbance of sample blank

#### 2.4.2. Hemolytic Activity

Hemolytic activity was assessed by adopting the procedure presented by Ven et al. 2012 [21]. Voluntary obtained human blood (10 mL in EDTA-Tube) was centrifuged at 2500 rpm for 10 min. Plasma was removed from red blood cells, and they were then thoroughly washed three times in a cooled PBS solution. RBCs (180 L) were combined with 20 L of the studied samples (20 mg/mL), and they were then incubated at 37 °C for 30 min. Incubated samples were centrifuged at 2900× *g* for 10 minutes. Supernatant (100 µL) was mixed with PBS (900 µL), and reading was noted at 576 nm on an ELISA reader. Positive control and negative control were also run in a similar manner, and percentage hemolysis was calculated by the following formula,

$$\%\;\mathrm{hemolysis}=\frac{\left(\mathrm{Absorbance}\;\mathrm{of}\;\mathrm{sample}-\mathrm{absorbance}\;\mathrm{of}\;\mathrm{negative}\;\mathrm{control}\right)}{\mathrm{absorbance}\;\mathrm{of}\;\mathrm{positive}\;\mathrm{control}}\times 100$$

## 3. Results and Discussion

### 3.1. Analysis of Data and Optimization

Within a limited number of experimental runs, a central composite design using response surface methods was used to study the specific interaction between formulative parameters (concentration of stabilizer and volume of antisolvent) on responses (particle size, zeta potential, and PdI). The formulative parameters and their responses are listed in Table 1.

After fitting these data, the DOE software 2.2 produced appropriate model equations including individuals and combined effect. *p* value was used to evaluate the importance of the quadratic model for the particle size, zeta potential, and polydispersity of nanosuspensions made using HPMC. A significant interaction between independent factors and responses is indicated by a *p* value less than 0.05. The results of the ANOVA show that all models were significant because the *p* value for each of the response parameters employed in this investigation was less than 0.05. The model equations for all responses are given below.

The model equation for particle size (Y_1_):

Y_1_ = +386.72 + 222.81 × 1 − 11.46 X_2_ − 91.50 × 1 × 2 + 467.47 × 1^2^ + 441.56 × 2^2^

The model equation for zeta potential (Y_2_):

Y_2_ = −10.26 − 0.75 X_1_ − 4.25 X_2_ − 0.49 X_1_ × 2 − 0.14 × 1^2^ + 1.14 × 2^2^

The model equation for PdI (Y_3_):

Y_3_ = +0.32 + 0.11 X_1_ − 0.031 X_2_ − 0.074 X_1_ × 2 + 0.15 × 1^2^ + 0.035 × 2^2^

A positive sign in the above equations indicated an effect that favors the optimization due to the synergistic effect, while a negative sign exhibited an antagonist or inverse relationship between the factors and responses.

### 3.2. Effect of Independent Factors on Particle Size, Zeta Potential, and PdI

**Figure 3:**
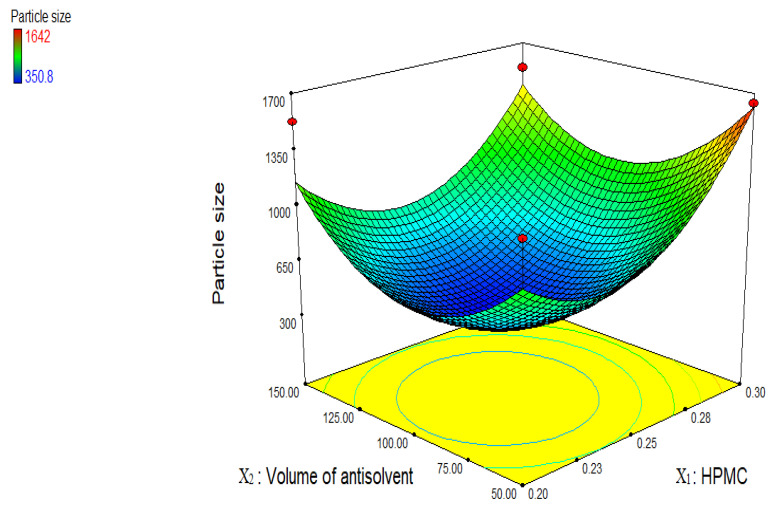
Three-dimensional graph showing the influence of independent variables on particle size of *R. serpentina* nanosuspensions using HPMC stabilizer.

An appropriate concentration of stabilizer is an important parameter during formulation of stable nanosuspension. Figure 3 displays a three-dimensional graph illustrating the impact of independent variables on particle size. Particle size decreased from 1528 to 350.8 nm when the quantity of HPMC was increased from 0.2 g to 0.25 g at a constant volume of antisolvent, then increased from 350.8 nm to 1642 nm when the amount of HPMC was increased from 0.25 g to 0.30 g. The following elements could be to blame for the decrease in particle size: a polymer called HPMC provides steric hindrance by forming a hydrodynamic boundary layer surrounding a nanosuspension and by adsorbing stabilizer to the surfaces of growing crystals, which hinders drug particle aggregation and growth. The drug-stabilizer system can be explained in terms of the hydrogen bonds that exist between the *Rauvolfia serpentina* drug particles and the copious amounts of hydroxyl groups in HPMC. The thickening of stabilizer layers on drugs that break or damage the protective layer around the nano particles could be the cause of the increase in drug particle size caused by increasing the concentration of HPMC [22,23]. Additionally, increasing HPMC content increases the viscosity of the solution, impairing the transmission of mechanical stirrer energy and preventing diffusion between the antisolvent (water) and solvent (ethanol) during nanoformulation precipitation. The ripening of Ostwald was the reason for the increasing particle size [18]. However, in this study, 0.25% *w/v* of HPMC concentration was found optimal to cover the entire drug surface and provides steric repulsion between nano-sized particles.

A sufficient amount of antisolvent also plays an important role to achieve nano-sized drug particles. As volume of antisolvent increased from 50 to 100 mL, particle size reduced from 1642 to 350.8 nm. It makes sense that a sufficient amount of antisolvent is needed for the stabilizer to cover the entire surface of the nanoparticles in order to prevent aggregation. Particle size increased from 350.8 to 1529 nm by increasing volume of antisolvent from 100 to 150 mL. There may have been an overabundance of antisolvent, which prevented the stabilizer from properly covering the particles to act as a barrier against aggregation, leading to increased particle size in the medication [24].

**Figure 4:**
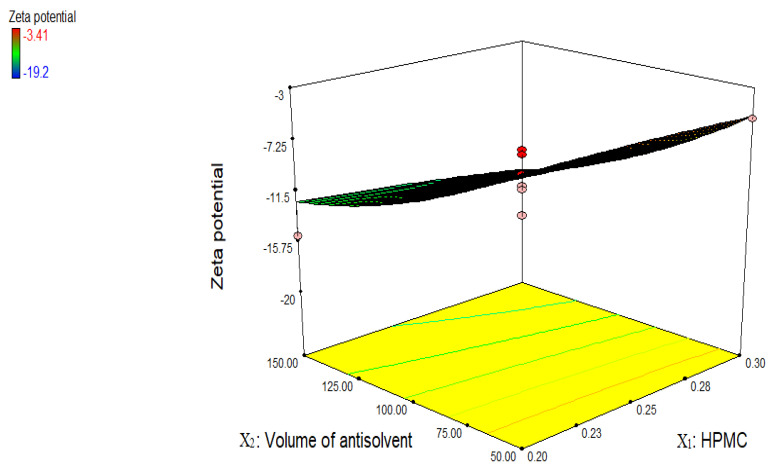
Three-dimensional graph showing the influence of independent variables on zeta potential of *R. serpentina* nanosuspensions prepared with HPMC.

Figure 4 shows effect of independent variables on zeta potential, which is an important parameter to check out the stability of prepared nanosuspension. Particle aggregation is less likely to occur if particles have enough zeta potential to produce steric or electric repulsion [18]. Steric stabilizers and electrostatic stabilizers with minimum zeta potential value ± 20 mV and ±30 mV, respectively, were recommended enough to stabilize the nanosuspension [13]. Zeta potential of nanosuspensions prepared with HPMC vary from −3.14 to −19.2 mV. All the formulations showed less zeta value except two (NPH 7 (−19.2 mV) and NPH 5 (−15.2 mV)). However, when polymers were utilized as a stabilizer, low reported zeta potential values did not signify low nanoparticle stability because the adsorbed layer of steric stabilizers increases the observed distance between the particle surface and shear plane, at which zeta potential was measured [25]. All the formulations have charged moieties that repel the drug particles and prevent agglomeration or Ostwald ripening; as a result, stable nanosuspension was developed. Since there were just two factors and 13 trials were produced using the CCD design, only five of the combination results were similar. The prevailing environmental temperature during the formation of the nanosuspension (even though efforts were made to keep it at the same level in each experiment) and, secondly, the possibility that the temperature changed during the characterization of these nanosuspensions are possible causes for the altered responses of the dependent variables.

**Figure 5:**
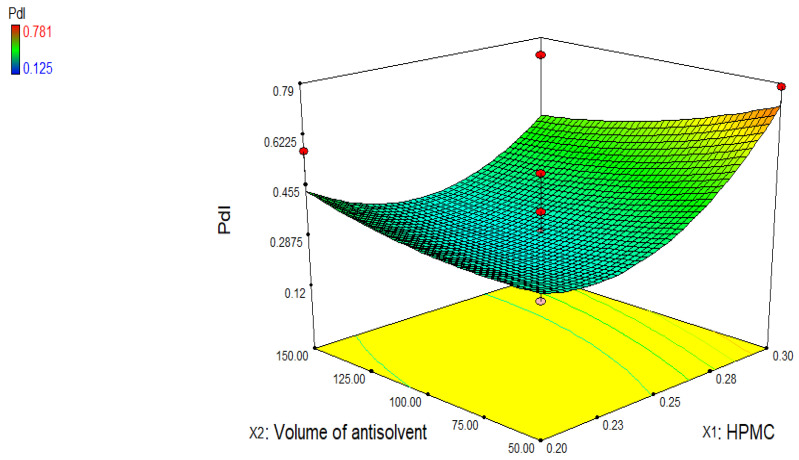
Three-dimensional graph showing the influence of independent variables on polydispersity index of *R. serpentina* nanosuspensions prepared with HPMC.

Similarly, Figure 4 shows effect of variables on polydispersity index (PdI), which is an important parameter to indicate the homogeneity of the particle size distribution in nanosystems. PdI value below 0.5 exhibits narrow size distribution of particles [26]. In the current study, PdI value of nanosuspensions prepared with HPMC ranges from 0.125 to 0.781 (Figure 5). The larger value of PdI (more than 0.5) represents that size of particles significantly differ from each other. It might be due to low stirring speed (3000 rpm) and time (6 h). Good PdI value can be achieved by increasing the speed of the mechanical stirrer or time more than 6 h.

**Figure 6:**
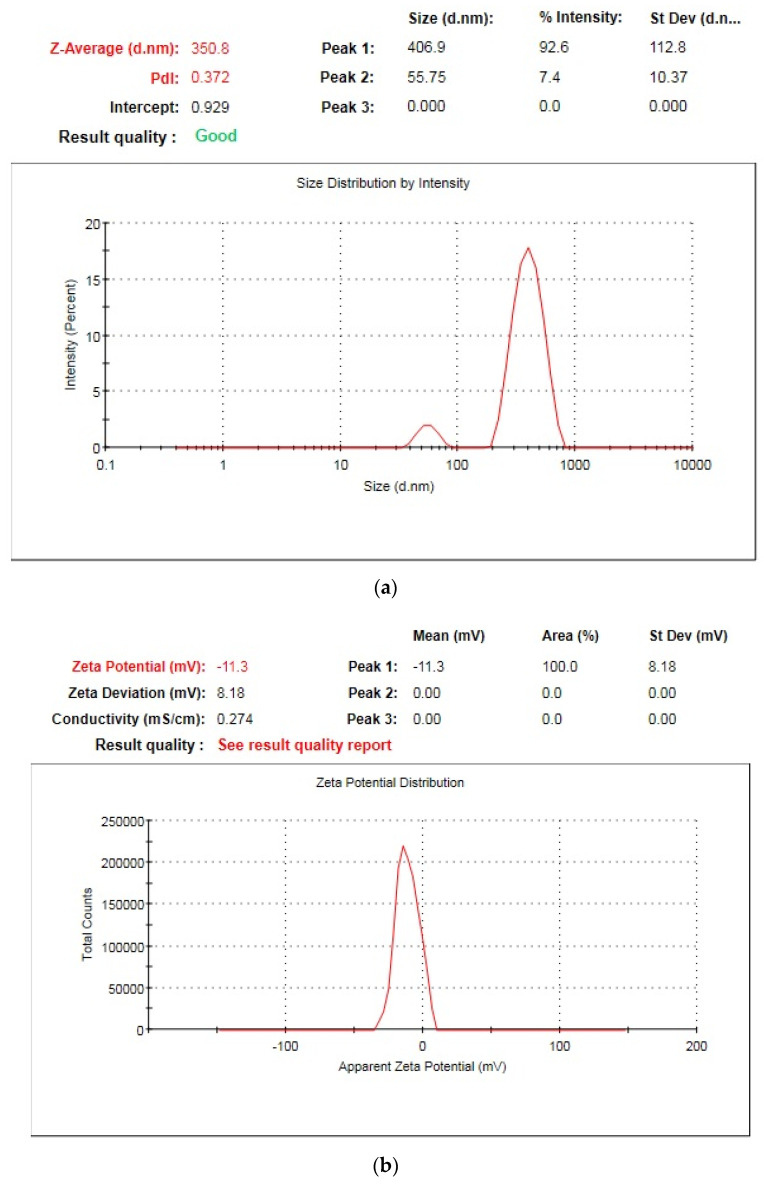
Graphical representation of optimized nanosuspension of *R. serpentina* for particle size and PdI (**a**) and zeta potential (**b**).

HPMC (hydroxy propyl methylcellulose) is a biodegradable natural cellulose polymer that is widely used in pharmaceutical drug delivery systems as a stabilizing agent. By adhering to the rising crystal face and enclosing the nanosuspension with a hydrodynamic boundary layer, the non-ionic polymer HPMC sterically prevents drug particle aggregation. In general, the stiffer the polymer chains are, the more growth inhibitory properties they will have. HPMC is a very rigid polymer due to the length of its backbone and side chains that also favors its ability as an inhibitor of growth [1]. Stabilization of nanosuspension can be explained on the basis of hydrogen bonding between drug particles and excess number of hydroxyl groups (−OH) on HPMC and thus had satisfactory affinity for nano-sized particles’ surfaces to inhibit agglomeration or Ostwald’s ripening [24]. In the present study, NPH9 (0.25% *w/v* in Table 1) selected as an optimized sample on the basis of particle size (350.8 nm), PdI (0.372), as shown in Figure 6a, and zeta potential (−11.3 mV), as shown in Figure 6b. This optimized sample was used for further biological investigations.

### 3.3. Scanning Electron Microscopy

**Figure 7:**
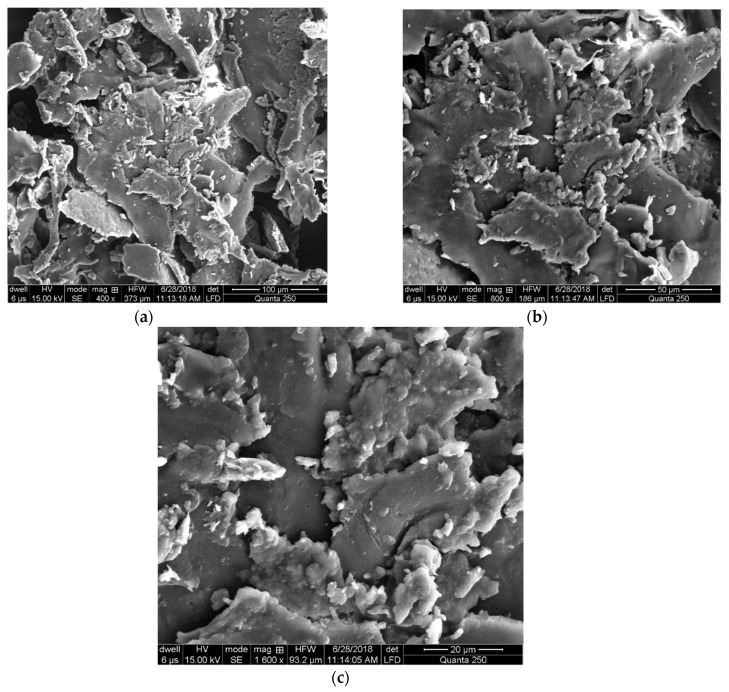
SEM images of optimized nanosuspension prepared with HPMC under different matrix at 400 (**a**), 800 (**b**), and 1600 (**c**) magnifications of optimized sample (0.25% *w*/*v* of independent variables).

The morphological properties of optimized *R. serpentina* nanosuspension prepared with HPMC was observed by using scanning electron microscopy. Scanning electron microscopy is a type of electron microscopy that scans the surface of a specimen with a focused beam of electrons. The electrons’ interactions with the specimen’s atoms result in signals that reveal its topography and composition. At 400, 800, and 1600 magnifications in the current investigation, the findings showed that rod-like particles were uniformly dispersed throughout the matrix, as illustrated in Figure 7a–c, respectively.

### 3.4. ACE Inhibitory Assay

**Figure 8:**
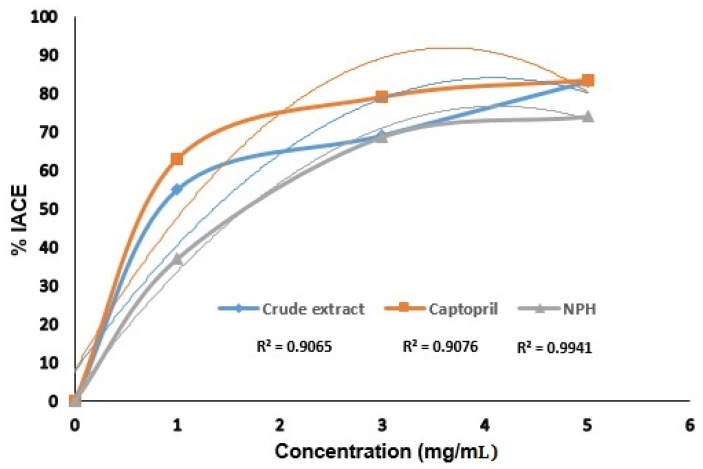
% Inhibition of angiotensin converting enzyme by crude extract and optimized nanosuspension (NPH = nanosuspension prepared with HPMC).

Angiotensin converting enzyme inhibitory (ACEI) potential of crude extract of *Rauvolfia serpentina* and optimized nanosuspension were studied at different concentrations as shown in Figure 8. Crude extract of *R. serpentina* and captopril revealed almost equal (83.011% and 83.33%) ACE inhibitory potential at 5 mg/mL. The inclusion of *Rauvolfia serpentina* flavonoids, which have higher ACE inhibitory capacity than the other tested samples, may account for the crude extract’s higher inhibitory potential. Through hydrogen bonding, the hydroxyl groups of flavonoids attach to ACE, changing the enzyme’s activity and specificity while preventing it from carrying out its intended task. More specifically, lone pairs of heterocyclic oxygen atoms assisted flavonoids in chelating or coordination of the zinc ion, which serves as the main active site of ACE. As a result, ACE was suppressed, which led to a reduction in blood pressure. The experimental data were determined to be best suited by polynomial second order regression model equations, as illustrated in Figure 8.

Additionally, nanosuspension made with HPMC demonstrated strong ACE inhibitory activity. It showed 73.99% ACE inhibition at 5 mg/mL concentration. It might be because HPMC contains large amounts of hydroxyl groups, which have the power to alter the properties of ACE by H-Bonding between the hydroxyl groups in nanosuspension made with HPMC and the active sites of ACE. Moreover, hydrophilic carrier HPMC (Hydroxy Propyl Methyl Cellulose) is most important in oral drug delivery systems. High swell-ability of HPMC is its main characteristic. Through interaction with water or gastric fluid, the HPMC’s morphology changed, allowing the drug to escape from the HPMC barrier by relaxing the structure’s backbone due to an excess of water [27]. Due to the nano-sized Rauvolfia serpentina drug particles and the presence of phenolic and flavonoids hydroxyl groups, the released medication performs its function as an ACE inhibitor by hydrogen bonding with ACE to inhibit ACE and ultimately cause a decrease in blood pressure. By blocking ACE and lowering blood pressure, flavonoids, phenolic substances, and peptides have been reported to have cardiac protective effects [3,28]. Hansen et al. 1996 [29] reported that flavonoids ACE inhibitory potential depends upon the position of the substituted hydroxyl groups (-OH). The ACE inhibitory potential of the hydroxyl groups close to the heterocyclic oxygen was better. Hetrocyclic oxygen and the presence of C-3, C-5, and C-7 hydroxyl groups were shared by all types of flavonoids studied. It was discovered that the presence of hydroxyl groups (−OH) at the C-7 and C-8 positions was effective to inhibit ACE [7].

### 3.5. Hemolytic Assay

The word hemolysis is used to indicate the breakdown of erythrocyte membrane with the discharge of hemoglobin (Hb) into plasma. Hemolytic activity of optimized nanosuspension and crude extract was tested in vitro on human erythrocytes. Table 2 lists the percentage of hemolysis for each sample. At 20 mg/mL, crude extract showed 4.27% hemolysis. Both the optimized nanosuspension and their negative controls did not exhibit any hemolysis when used at the same concentration. Entrapping the medication in stabilizer and nanosizing *Rauvolfia serpentina* could both inhibit the herb’s hemolytic activity [21]. These results provided support for nanosuspension non-toxicity.

## 4. Conclusions

Due to its advantages in reducing the size of medication particles, nanosuspension is a cutting-edge method in pharmaceutical science. In the present investigation, ethanol was used as the solvent and distal water as the antisolvent in the antisolvent precipitation procedure to create *Rauvolfia serpentina* nanosuspensions in the presence of the stabilizer HPMC. To create an optimal sample, the impact of stabilizer concentration and antisolvent volume on particle size, zeta potential, and PdI through CCD-RSM was investigated. The optimized nanosuspension had a particle size of 350.8 nm, a zeta potential of −11.3 mV, and a PdI value of 0.372. It was made with HPMC (NPH9) and 0.25% *w/v* of independent variables. The SEM analysis showed rod shape particles in nanosuspension prepared with HPMC. A further in vitro biological study found that crude extract has greater potential to block the angiotensin-converting enzyme than nanoformulations. The %IACE was in the following order Captopril (83.33%) > crude extract (83.11%) > optimized nanosuspension prepared with HPMC (73.99%) at 5 mg/mL. The real behavior of nanosizing for inhibiting angiotensin converting enzyme was suggested by in vivo experiments. Due to the drug particles being trapped by the stabilizer, the findings of the hemolytic activity revealed that the nanosuspension was non-hemolytic towards red blood cells, while crude extract showed 4.31% hemolysis, making it unsafe for use with human erythrocytes. The study’s findings, taken together, suggest a methodical approach for examining the effects of crucial formulation and processing variables when creating nanosuspensions to increase the solubility and rate of dissolution of less soluble active ingredients in *Rauvolfia serpentina* using the antisolvent precipitation technique.

## Figures and Tables

**Table 1:** Experimental design along with responses for *R. serpentina* nanosuspensions prepared with HPMC (NPH) by using CCD-RSM.

Sr. No.	Concentration of Stabilizer (g)	Volume of Antisolvent (mL)	Particle Size (nm)	Zeta Potential (mV)	PdI
NPH1	0.20	50	1275	−3.41	0.322
NPH2	0.30	50	1642	−5.47	0.781
NPH3	0.18	100	623.8	−2.55	0.438
NPH4	0.25	171.71	990.2	−15.2	0.139
NPH5	0.20	150	1528	−10.4	0.572
NPH6	0.32	100	1624	−8.93	0.622
NPH7	0.30	150	1529	−19.2	0.724
NPH8	0.25	29.29	1154	−0.362	0.444
NPH9	0.25	100	350.8	−11.3	0.372
NPH10	0.25	100	441.1	−10.99	0.3
NPH11	0.25	100	405	−11	0.5
NPH12	0.25	100	355.8	−8	0.125
NPH13	0.25	100	380.9	−10	0.29

**Table 2:** %age hemolysis of optimized *R. serpentina* nanosuspensions and crude extract.

Samples	%Age Hemolysis
Crude extract	4.27 ± 0.01
HPMC	−11.84 ± 0.01
NPH	−1.283 ± 0.001
Triton X-100	57.91 ± 0.01

NPH = nanosuspension Prepared with HPMC.

## Data Availability

Not applicable.

## Abbreviation

ACE	Angiotensin converting enzymes
HPMC	Hydroxyl propyl methyl cellulose
SEM	Scanning electron microscopy
RAAS	Renin–angiotensin–aldosterone system
DLS	Dynamic light scattering zetasizer
DOE	Design of expert
RSM	Response surface methodology
NPH	Nanosuspension prepared with HPMC
ACEI	Angiotensin converting enzymes inhibition
PdI	Polydispersity index
